# RNAalifold: improved consensus structure prediction for RNA alignments

**DOI:** 10.1186/1471-2105-9-474

**Published:** 2008-11-11

**Authors:** Stephan H Bernhart, Ivo L Hofacker, Sebastian Will, Andreas R Gruber, Peter F Stadler

**Affiliations:** 1 Bioinformatics Group, Department of Computer Science, and Interdisciplinary Center for Bioinformatics, University of Leipzig, Härtelstrasse 16-18, D-04107 Leipzig, Germany; 2 Institute for Theoretical Chemistry, University of Vienna, Währingerstrasse 17, A-1090 Vienna, Austria; 3 Bioinformatics Group, Department of Computer Science, University of Freiburg, Georges-Köhler-Allee, Geb. 106, D-79110 Freiburg, Germany; 4 RNomics Group, Fraunhofer Institut for Cell Therapy and Immunology (IZI) Perlickstrasse 1, D-04103 Leipzig, Germany; 5 The Santa Fe Institute, 1399 Hyde Park Rd., Santa Fe, New Mexico

## Abstract

**Background:**

The prediction of a consensus structure for a set of related RNAs is an important first step for subsequent analyses. RNAalifold, which computes the minimum energy structure that is simultaneously formed by a set of aligned sequences, is one of the oldest and most widely used tools for this task. In recent years, several alternative approaches have been advocated, pointing to several shortcomings of the original RNAalifold approach.

**Results:**

We show that the accuracy of RNAalifold predictions can be improved substantially by introducing a different, more rational handling of alignment gaps, and by replacing the rather simplistic model of covariance scoring with more sophisticated RIBOSUM-like scoring matrices. These improvements are achieved without compromising the computational efficiency of the algorithm. We show here that the new version of RNAalifold not only outperforms the old one, but also several other tools recently developed, on different datasets.

**Conclusion:**

The new version of RNAalifold not only can replace the old one for almost any application but it is also competitive with other approaches including those based on SCFGs, maximum expected accuracy, or hierarchical nearest neighbor classifiers.

## Background

Unbiased surveys of the transcriptomes of higher eukaryotes by multiple techniques ranging from tiling arrays and short-read sequencing to large-scale sequencing of full-length cDNAs have dramatically changed our perception of genome organization: At least 90% of the mammalian genomes are transcribed, the vast majority of this transcription is non-protein-coding, and there is mounting evidence that a significant fraction of the non-coding transcripts are functional [1,2]. The investigation of non-coding RNAs has thus developed into a focal topic in molecular biology and bioinformatics alike. Most of the ancient house-keeping RNAs (tRNAs, rRNAs, snRNAs, snoRNAs) and many of the newly discovered regulatory RNAs, including microRNA precursors, form evolutionarily well-conserved secondary structures, reviewed e.g. in [3]. These structures are tightly linked to the molecules' functions. It is therefore a core task in RNA bioinformatics to compute in particular the consensus structures of evolutionarily conserved RNAs.

It has long been known that the accuracy of thermodynamic structure predictions for individual sequences is rather limited. On the other hand, computing the *consensus structure *common to several related RNA sequences can drastically improve the prediction [4]. The conceptually most elegant approach towards consensus structure prediction is to solve the alignment and the structure prediction problem simultaneously. The Sankoff algorithm [5] provides a solution that is practically applicable and has been implemented in various variants including dynalign [6], stemloc [7], foldalign [8], LocARNA [9] or consan [10]. Still, these approaches are computationally too expensive for large-scale routine applications. One basic alternative is to first compute structures for the individual sequences and then to align these sequences taking into account the structural information. This can be achieved in different ways using sequence-based (e.g. stral [11]), tree-based [12,13], or Sankoff-style alignment algorithms [14]. Alignment-free approaches include RNAspa [15] and consensus shapes [16].

A large group of methods pre-supposes a (sequence) alignment. Most methods of this type use the alignment to super-impose predicted structures to global [17,18] or local structures [19]. RNAalifold [4], on the other hand, in essence averages the contributions of the standard Turner energy model [20] according to a given alignment A and then solves the thermodynamic folding problem w.r.t. these averaged energies. A special case is the ConStruct package [21], which besides acting as a front-end for several prediction tools provides an interface for changing RNA alignments using expert knowledge.

## Methods

### Original RNAalifold

The original RNAalifold approach combines a thermodynamic energy minimization [22] with a simple scoring model to assess evolutionary conservation. Both an energy minimization and a partition function version are implemented in the Vienna RNA package [4]. Energy minimization uses the following recursions:

$$\begin{array}{lll} F_{i,j} & = & \min\left(F_{i+1,j},\underset{i<k\leq j}{\min}C_{i,k}+F_{k+1,j}\right)\\ C_{i,j} & = & \beta \gamma (i,j)+\\ & + & \min\{\begin{array}{l} \sum_{\alpha \in A}ℌ(i,j,\alpha )\\ \underset{i<k<l<j}{\min}\left(\sum_{\alpha \in A}J(ij,kl,\alpha )+C_{k,l}\right)\\ \min_{i<k<j}\left(M_{i,k}+M_{k+1,j}^{1}+a\right) \end{array}\\ M_{i,j} & = & \min\{\begin{array}{l} M_{i+1,j}+c\\ \underset{i<k<j}{\min}C_{i,k}+M_{k+1,j}+b\\ M_{i,j}^{1} \end{array}\\ M_{i,j}^{1} & = & \min\left(M_{i,j-1}^{1}+c,C_{i,k}\right) \end{array}$$

As in single-sequence folding, the arrays *F*_*ij*_, *C*_*ij*_, *M*_*ij*_, and $M_{ij}^{1}$ hold, for every sub-sequence from *i *to *j*, the energies of the optimal folds of unconstrained structures, of structures enclosed by (*i*, *j*) base pairs, of multi-loop components, and of multi-loop components with a single branch, respectively [23]. The Turner energy parameters for hairpin loops delimited by *alignment *positions *i *and *j *in sequence *α *∈ A are denoted by ℌ(*i*, *j*, *α*); similarly ℑ(*ij*, *kl*, *α*) encodes the energies of interior loops including stacked base pairs. Multi-loops are modeled by a linear model with a "closing" contribution a, and contribution b and c for each branch and unpaired position, respectively. Note that these values are the tabulated single-sequence parameters multiplied by the number *N *= |A| of aligned sequences, since the recursion above computes the sum of the folding energies. RNAalifold modifies the energy model by introducing a (base pair) conservation score *γ*(*i*, *j*) that evaluates the corresponding alignment columns w.r.t. evidence for base pairing. In [4], we used

(1) $$\gamma^{\prime }(i,j)=\frac{1}{2}\sum_{\begin{array}{c} \alpha ,\beta \in A\\ \alpha \neq \beta \end{array}}\{\begin{array}{ll} h(\alpha_{i},\beta_{i})+h(\alpha_{j},\beta_{j}) & \text{if }(\alpha_{i},\alpha_{j})\in ℬ\\ & \wedge (\beta_{i},\beta_{j})\in ℬ\\ 0 & \text{otherwise} \end{array}$$

where the Hamming distance *h*(*a*, *b*) = 0 if *a *= *b *and *h*(*a*, *b*) = 1 if *a *≠ *b *and ℬ = {*AU*, *UA*, *CG*, *GC*, *GU*, *UG*} is the set of possible base pairs. The full covariation score *γ *also includes penalties for sequences in which the (*i*, *j*) base pair cannot be realized:

(2) $$\gamma (i,j)=\gamma^{\prime }(i,j)+\delta \sum_{\alpha \in A}\{\begin{array}{l} 0\text{ if }(\alpha_{i}\alpha_{j})\in ℬ\\ 0.25\text{ if }\alpha_{i}\wedge \alpha_{j}\text{ are gaps}\\ 1\text{ otherwise} \end{array}$$

Potentially paired columns, in which less than a user-defined number or fraction of sequences can form the pair, are considered to be forbidden. RNAalifold therefore predicts the structure common to *most *of the sequences in an alignment. A prediction for a single molecule that is consistent with the consensus structure can be obtained by using the result of RNAalifold as a constraint for single molecule folding. Both mfold [22] and RNAfold [24] can be used for this purpose.

The purpose of this contribution is to explore several avenues for improving the performance of RNAalifold. Intuitively, there are two leverage points: (1) the details of the energy evaluations in the presence of gaps, and (2) the rather *ad hoc *covariance bonuses and penalties.

### Improved Energy Evaluation

**Figure 1:**
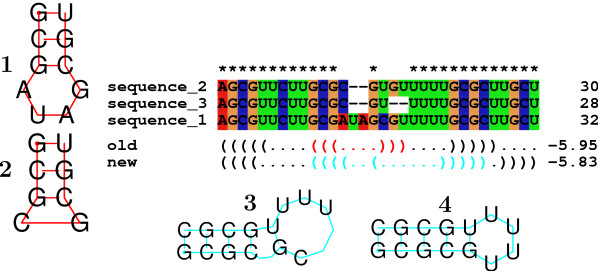
**Possible results of treating gaps as bases.** The consensus structure of the alignment in the middle is predicted once with gaps treated as if they were bases (old), and once by removing them before computing the energies (new). The predicted structures (highlighted in red) are shown to the left. As can be seen in **1**, sequence 1 can form a perfect hairpin. In **2**, the sterically impossible hairpin for the other two sequences is shown. Two of the three sequences cannot form the predicted structure. On the other hand, the new version of RNAalifold predicts a stem that has a bulge (**3**), but only in one sequence, the other two sequences can form the perfect stem shown in **4**.

The 2002 implementation of RNAalifold uses a very simplistic way of treating gaps in order to save computational resources: gaps within unpaired regions are simply ignored, because then only alignment positions appear as indices and loop sizes, for instance, do not need to be evaluated separately for every sequence. This can, however, distort the energetics in particular if there are many gaps, and in extreme cases can lead to the inclusion of unrealistically short hairpins, see Figure 1. A second source of error is that gaps do not contribute to the dangling end energies in this setting.

The new implementation thus evaluates ℌ(*i*, *j*, *α*) and ℑ(*ij*, *kl*, *α*) by first mapping the alignment indices back to the positions in *α*. Then the correct energy parameters according to the Turner model are retrieved. In the same way, the handling of dangling ends is fixed. In practice, this is achieved by introducing three arrays of dimension *N *× *n*, where *n *is the length of the alignment of *N *sequences. For each sequence *α *and each alignment position, these arrays hold the 5' neighboring base, the 3' neighboring base, and the position in the original sequence. Since in typical applications we have *N *≪ *n*, this does not significantly change memory consumption. Still, the problem remains that in some sequences hairpin loops with less than three unpaired positions may arise. We penalize these sequences with a contribution of the same order of magnitude as that of non-canonical base pairs. From here on, we will refer to this "gap free" energy computation as the "new RNAalifold".

#### Energy Parameters

Instead of the usual Turner energy parameters, one may use other parametrizations. Andronescu *et al*. [25] introduced energy parameters that increase the performance of single stranded RNA folding, with striking results in particular on ribosomal RNAs. We found, however, that they provide no significant over-all performance gain for RNAalifold on the broad range of datasets we used to assess performance (see section *Performance Evaluation *below). The results obtained for Andronescu's energy parameters, together with those of other unsuccessful attempts to increase performance, are tabulated in the additional file 1.

#### Sequence Weighting

In practice, many input alignments have a very unbalanced distribution of sequences. Often most sequences are very closely related and outweigh one or a few divergent ones. In this case it seems appropriate to down-weight the influence of closely related sequences [26] similar to the weighted sum of pairs score frequently used for multiple alignment. The problem with this approach is that distant sequences receive the highest weights, but are also more likely to be misaligned, and hence a rational weighting scheme will also increase the impact of alignment errors.

One can try to minimize this effect by dividing the score of RNAalifold in two parts, one which does not contain the outliers, thus scoring a smaller alignment, and one which contains all sequences. If the smaller alignment scores significantly better than the complete one, one can assume that the divergent sequence is either misaligned or at least does not share the consensus structure. At present, we have not been able to devise a fail-safe automatic procedure to identify these cases. Since sequence weighting leads to a significant increase in CPU time because the weighting has to be introduced in the inner-most loop of the energy evaluation, we have decided against including the weighting option into the public version of RNAalifold.

### Improving the Evaluation of Sequence-Covariation

#### RIBOSUM Matrices

The covariance term *γ' *of the old RNAalifold implementation is based on qualitative arguments only. A more quantitatively sound approach is to use scoring matrices akin to the RIBOSUM scheme [27]. As a training set, we selected 13,500 sequences in total from the about 20,000 sequences in the SSU alignment of the European Ribosomal RNA Database [28], which are available in the DCSE file format. When reading in the DCSE format, one needs to correctly assign helix numbers to concrete helices of the sequences. In some cases, this assignment could not be done in an automated way. Avoiding possible mis-assignments, such base pairs were ignored in the computation. We also kept only sequences with less than 5% undetermined nucleotides and at least 50% of the maximum possible number of base pairs. This set was clustered using single linkage clustering to determine clusters where the sequence identity between different clusters is ≤ *P*. For each cutoff value *P *we determined the frequencies *f*(*ac*) of nucleotides of type *a *and *c *being aligned and *f*(*ab*; *cd*) of base pairs of type *ab *and *cd *being aligned in sequences that are within different clusters. Besides being more different than *P*, the sequences had to have at least a sequence identity of *Q*. For each pair *Q*, *P*, we define the modified RIBOSUM scores as the log-odds scores

(3) *R*(*ab*, *cd*) = log
                        (*f*(*ab*;
                        *cd*)/*f*(*ac*)*f*(*bd*))

In practice, we vary *P *and *Q *in steps of 5% sequence identity and obtain altogether 99 matrices. Note that this procedure is somewhat different from the approach reported in [27]. The frequencies can be determined either for all base pairs including the non-canonical ones or restricted to the six types of canonical base pairs. Only the latter version has proved useful in our context, and will be referred to as RIBOSUM in the following.

The covariance term is computed as

(4) $$\gamma^{\prime }(i,j)=\frac{1}{2}\sum_{\begin{array}{c} \alpha ,\beta \in A\\ \alpha \neq \beta \end{array}}xR(\alpha_{i}\alpha_{j};\beta_{i}\beta_{j}),$$

i.e., the RIBOSUM matrices replace the Hamming distances *h*(*α*_*i*_, *β*_*i*_) + *h*(*α*_*j*_, *β*_*j*_), and are scaled by a factor *x *so that the entries are in the same range as the entries of the Hamming distance matrix. In order to determine which matrix to use, we determine the minimum *q *and maximum *p *sequence identity in the alignment and select the RIBOSUM matrix with smallest *P *and *Q *so that *p *≤ *P *and *q *≤ *Q*.

RNAalifold uses two parameters to fine-tune the impact of the covariance score. The first parameter, *β*, controls the influence of the covariance score *γ' *relative to the total folding energy. The second one, *δ*, weights the impact of non-standard pairs. The old default value for both parameters is 1.

Simply leaving them as they are would lead to a large change in the balance between the thermodynamic and the covariance score. In the old RNAalifold program, less than 10% of the total score is derived from the covariance score. If *β *and *δ *were kept at 1, this fraction would increase to more than 50%. This would presumably overemphasize covariance over thermodynamics. To find appropriate values for *β *and *δ*, we use *k*-fold cross validation, with *k *= 11 on the CMfinder-SARSE benchmark dataset described below.

#### Pfold-like Scoring

Inspired by the approach used in Pfold, we also tested a covariance scoring based on an explicit phylogenetic model. More precisely, we used the log-odds ratio of the probabilities of a base pair given a tree and the alignment, and the product of the corresponding probabilities of unpaired bases given the same tree and alignment [29]. A neighbor joining tree computed from the distances measured within the alignment was used. The probabilities were then computed from this tree using the Pfold rate matrices. This ansatz, however, did not result in more accurate predictions. Therefore, it was not included into RNAalifold.

### Additional features

In addition to increasing the performance, additional functionalities are included in the new RNAalifold software.

#### Centroid structure

The partition function computation now includes the computation of the *centroid structure*, which is defined as the structure with minimal mean base pair distance to all the structures of the ensemble:

(5) $$d(S)=\sum_{i,j\in B}\{\begin{array}{ll} 1-(p(i,j)) & \begin{array}{cc} \text{if} & i,j\in B(S) \end{array}\\ p(i,j) & \text{else} \end{array}$$

Here, *d*(*S*) is the distance of a structure to the ensemble, *B *denotes the set of all possible base pairs in the ensemble, *B*(*S*) is the set of all base pairs of structure *S*, and *p*(*i*, *j*) is the probability of the base pair *i*, *j *in the ensemble. It can easily be seen that the structure with minimal *d*(*S*) is the structure that contains all base pairs with a probability greater than 0.5. This centroid structure can be seen as the single structure that best describes the ensemble [30]. The centroid structure usually contains less base pairs than the minimum free energy structure, and is therefore less likely to contain false positives.

#### Stochastic Backtracking

When trying to find out about statistical features of the structure ensemble other than base pair probabilities, it is sometimes of interest to compute a sample of suboptimal structures according to their Boltzmann weights. This can be achieved efficiently using so-called stochastic backtracking. In this variation of the standard backtracking scheme, one uses the matrices of the partition function computation to determine the probability of base pairs or unpaired bases that are included in the structure instead of choosing the alternative with the minimum free energy at each step. The principle of stochastic backtracking in RNA folding has been used already in [31] for the generation of uniformly distributed random structures. Later, sfold [32] and the Vienna RNA Package [24] also implemented energy-weighted variants. These implementations differ from the original algorithm only by the inclusion of the Boltzmann factors of the loop energy contributions instead of treating all structural alternatives with equal weight. The generalization of the stochastic backtracking algorithm to consensus folds is straightforward. See additional file 2 for a detailed description. Stochastic backtracking is now implemented in the RNAalifold software.

### Performance Evaluation

A trusted set of aligned sequences with corresponding structures is needed in order to evaluate the performance of consensus structure prediction tools. Most papers on this topic use some subset of the Rfam [33]. However, the structures and alignments contained in Rfam pose several problems. The database consists of a large number of snoRNAs (more than 30% of the alignments) and micro RNAs (about 7%). Furthermore, many of the Rfam entries contain short sequences that can only form simple one stem structures. A serious problem is the fact that many of the Rfam structures are predictions, some of which were created by the very programs that are to be tested. Not even all of the structures flagged as published within the database have been experimentally derived. Mostly because of this reasons, only 19 of the more than 600 Rfam families are contained in RNA STRAND [34], a recently created, curated database of high quality single RNA secondary structures.

We therefore chose several different datasets for performance evaluation. In addition to the complete Rfam (version 8.1) seed alignments, we use here the CMfinder-SARSE subset compiled from [35,36], which contains 44 high quality seed alignments (also used in the recent PETfold paper [37]), the seeds of 19 Rfam families contained in RNA STRAND, and the dataset of KNetFold [38]. A list of these Rfam subsets can be found in the additional file 3 or including links in the online supplement.

The script refold.pl of the Vienna RNA package is used to remove gaps and non-standard base pairs from the RNAalifold predictions. The resulting structure is compared to the reference structure. For each alignment only the first sequence is used for performance evaluation to avoid a bias from the unequal sizes of the aligned sequence sets. As performance measure we use the Mathews correlation coefficient (MCC) as introduced in a previous benchmark [39]: Base pairs that are not part of the reference structure are counted as false positives only if they are inconsistent with the reference structure, while they are ignored if they can be added to the reference structure. Thus additional stems and elongated stems are not penalized. While this is a physically reasonable way to compute the MCC, the question of comparability might arise. To address this, we also used the more simple way of defining false positives as all base pairs predicted that were not part of the reference structure, and called it "other MCC".

For the comparison procedure, we used the web-servers of Pfold [29] and KNetFold. In case the Rfam seed alignment contained more than 40 sequences, only the first 40 were used; all-gap columns were removed from such alignments. The McCaskill-MEA software McC_mea [17] was installed locally. The predictions were also filtered with refold.pl before scoring.

In order to evaluate the dependence on the alignment quality, we also realigned the Rfam alignments of the CMfinder-SARSE dataset using Clustal [40], and then proceeded as described above. Furthermore, we also computed the MCC for all Rfam seed alignments for those programs that can be run locally (i.e. the RNAalifold variants and McC_mea).

The RNAalifold algorithm has been extensively used for the prediction of thermodynamically stable and/or evolutionary conserved RNAs [41-43]. The AlifoldZ program [41] evaluates stability and structural conservation at the same time simply by comparing the consensus free energy of an alignment to the consensus free energies of a large number of randomly shuffled alignments, relying entirely on RNAalifold. RNAz [42], on the other hand calculates two separate scores for stability and conservation. Structural conservation is assessed by means of the folding energy based structure conservation index (SCI). Here, the consensus energy is set in relation to the mean free energies of the single sequences. The lower bound of the SCI is zero, indicating that RNAalifold is not able to find a consensus structure, while a SCI close to one corresponds to perfect structure conservation. Here, we investigate whether the improved performance of RNAalifold in terms of correctness of the predicted structure can also improve the performance of ncRNA gene finders.

In order to evaluate the performance of AlifoldZ and the SCI, we re-consider a sub-set of the test-set used in a previous benchmark [44]. As usual, we compute ROC curves to determine our ability to discriminate between truly conserved alignments and randomized controls. For simplicity, only the area under the ROC curve (AUC) is reported below as a measure of the discrimination power.

## Results and Discussion

### Predicting consensus structures

**Figure 2:**
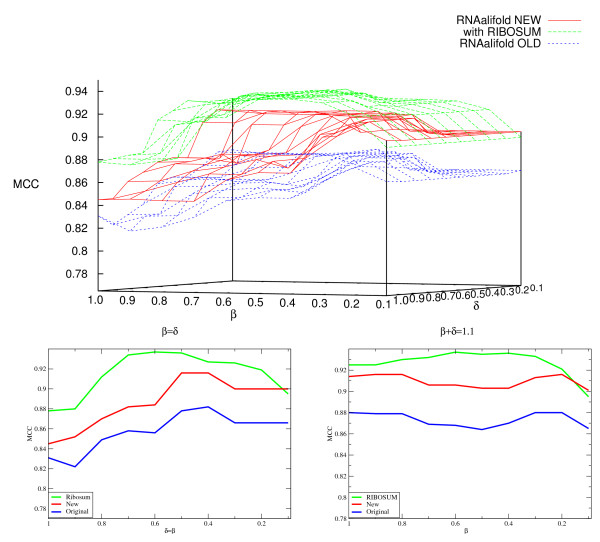
**MCC on the CMfinder-SARSE dataset as a function of the *β *and *δ *parameters.** It can be seen that except for *β *= 1.0, using RIBOSUM Matrices improves the performance of the new RNAalifold, which is in turn always better than the 2002 (old) variant. Furthermore, for the RIBOSUM variant, the size of the plateau, i.e. the subset of parameters with a MCC ≥ 0.93 is quite big, containing 36 of 100 combinations of parameters (80 are ≥ 0.9, 21 are ≥ 0.935 and 6 are 0.937). **Top**: 3d-plot of the MCC against the parameters *β *and *δ*. **Bottom**: Vertical section along the diagonals *β *= *δ *and *δ *+ *β *= 1.1.

**Figure 3:**
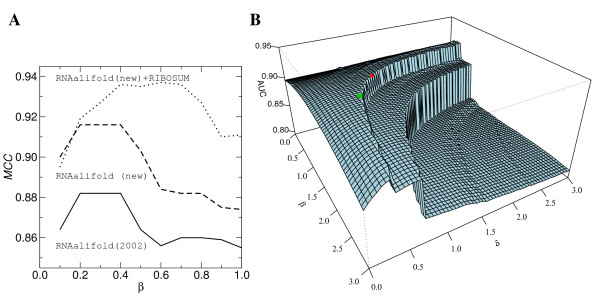
**Dependence of RNAalifold on the weights *β *and *δ*.A**: For all three RNAalifold variants, the accuracy of the structure prediction, measured here as MCC for the CMfinder-SARSE dataset (Table 1), depends on the weight *β *of the covariance term (*δ *= 0.6). **B**: The AUC value for the SCI computation also depends strongly on the values of *β *and *δ*. The green square indicates the optimal parameters (*β *= 1.55, *δ *= 0.6), the red dot is the default (1, 1). As the default is close to the maximum, there is little room for improvement.

We first compared the new implementation of RNAalifold with the 2002 version. As shown in Figure 2, the proper treatment of gaps in the new version leads to a consistently improved accuracy. The data also shows that the covariance contribution in the 2002 version was too large. Using RIBOSUM matrices instead of the naïve Hamming distance score substantially increases the beneficial effect of the covariance score. However, if the same parameters as in the original RNAalifold were used, the relative portion of the covariance term within the score would be greater than the thermodynamic score. We remark that for large values of *β*, where the covariance contributions dominate, the performance becomes much worse than for a purely thermodynamic energy computation (data not shown). As a new default, we therefore use *β *= 0.6 and *δ *= 0.5. Still, the portion of the covariance term in the combined energy term is much higher (about 44%) in the RIBOSUM than in the other RNAalifold variants (about 7%). We want to remark that with the exception of very low *β*, the performance of the RIBOSUM variant always exceeds the performance of the new variant without RIBOSUM, which in turn always performs better than the 2002 variant of RNAalifold (see Figures 2 and 3).

Table 1 summarizes the comparison of the consensus structure predictions for five alignment-based programs on the CMfinder-SARSE dataset. The new RNAalifold with RIBOSUM matrices often yields perfect predictions and appears to have a good worst case performance: the smallest observed MCC is 0.64, and in this case the input alignment is clearly flawed, see additional file 4.

**Table 1:** Results on the CMfinder-SARSE dataset

RNA	#seq	MPI	RIBOSUM	RNAalifold	Pfold	KNetFold	McC_mea
Antizyme_FSE	13	87	**1.000**	**1.000**	**1.000**	**1.000**	**1.000**
ctRNA_pGA1	15	72	**1.000**	**1.000**	**1.000**	0.976	**1.000**
Entero_5_CRE	160	84	**1.000**	0.848	0.478	**1.000**	0.942
Entero_CRE	56	81	**1.000**	0.736	**1.000**	0.953	0.953
GcvB	17	64	**0.939**	0.799	0.889	**0.939**	0.921
glmS	11	60	**0.986**	0.972	0.972	0.809	0.837
HACA_sno_Snake	22	90	0.871	0.407	0.414	**0.915**	0.884
HCV_SLIV	110	89	**1.000**	0.922	**1.000**	**1.000**	0.961
HDV_ribozyme	15	95	**0.953**	-0.015	0.590	0.460	0.460
HepC_CRE	52	87	**1.000**	0.962	**1.000**	**1.000**	**1.000**
Histone3	64	78	**1.000**	**1.000**	**1.000**	**1.000**	**1.000**
Hsp90_CRE	4	98	0.855	0.855	0.413	0.867	**0.874**
IBV_D-RNA	10	96	**1.000**	0.928	0.928	**1.000**	**1.000**
Intron_gpII	114	54	**1.000**	0.779	**1.000**	**1.000**	**1.000**
IRE	39	63	**1.000**	0.938	**1.000**	**1.000**	0.938
let-7	14	73	**1.000**	0.979	**1.000**	**1.000**	0.957
lin-4	9	73	**1.000**	0.973	**1.000**	**1.000**	**1.000**
Lysine	43	49	**0.990**	0.918	0.960	**0.990**	**0.990**
mir-10	11	67	**0.973**	0.888	0.916	**0.973**	**0.973**
mir-194	4	79	0.870	0.849	**1.000**	0.866	0.698
mir-BART1	3	93	0.977	0.977	0.861	**1.000**	0.977
nos_TCE	3	90	0.975	0.975	0.951	**1.000**	0.975
Purine	22	56	0.945	0.917	**1.000**	0.945	0.945
Rhino_CRE	12	72	0.734	0.734	0.680	**0.974**	0.756
RNA-OUT	4	96	0.775	0.775	**0.834**	0.740	0.775
rncO	6	80	0.903	**0.923**	0.668	0.896	0.825
Rota_CRE	14	86	**1.000**	0.764	0.682	0.099	-0.011
s2m	38	79	0.739	**1.000**	0.774	0.652	0.861
SCARNA14	4	67	**0.969**	0.748	-0.005	0.532	0.777
SCARNA15	3	96	**1.000**	**1.000**	0.601	0.971	0.925
SECIS	63	43	0.941	0.813	0.943	**0.971**	0.813
SNORA14	3	92	0.944	0.944	0.853	**0.959**	0.869
SNORA18	6	79	0.913	0.503	0.702	**0.971**	0.893
SNORA38	5	84	0.759	0.743	**0.858**	0.410	0.734
SNORA40	7	80	**0.962**	**0.962**	0.704	0.948	0.920
SNORA56	4	97	0.816	**0.922**	0.446	0.779	0.741
SNORD105	2	89	**1.000**	**1.000**	-0.007	0.648	0.971
SNORD64	3	94	**1.000**	0.539	0.539	0.661	-0.014
SNORD86	6	82	**0.641**	-0.012	-0.007	0.511	0.000
snoU83B	4	87	**0.927**	**0.927**	0.846	0.895	**0.927**
TCV_H5	3	97	**1.000**	**1.000**	0.685	**1.000**	**1.000**
TCV_Pr	4	95	**1.000**	**1.000**	0.688	**1.000**	**1.000**
Tymo_tRNA-like	28	64	**1.000**	0.916	**1.000**	0.973	**1.000**
ykoK	36	61	0.856	0.756	**0.906**	0.841	0.794

mean			**0.937**	0.831	0.765	0.866	0.837

Performance comparisons on the CMfinder-SARSE dataset. We list the MCC for different alignments. Best performance **bold**.

In Table 2, the performance of the same five programs on the RNA STRAND-Rfam dataset is shown. This curated dataset, in contrast to the other datasets we used, has many pseudo-knotted structures (6) and only 2 of the 19 alignments have simple one-stem structures. In this regard, it is a good extension to our other datasets. While the total MCCs of all programs are lower, again the RIBOSUM variant of RNAalifold outperforms the other programs – however, on this dataset, the centroid structure computed using RIBOSUM RNAalifold has the best performance, with an MCC of 0.794. For this table, KNetFold was run using the "check pseudoknots" option. Still, it only correctly predicted a part of a single pseudo-knot.

**Table 2:** Results on the RNA STRAND-Rfam dataset

RNA	comment	RIBOSUM	RNAalifold	Pfold	KNetFold	McC_mea
7SK		**0.507**	0.456	0.292	0.429	0.306
bicoid_3		**0.949**	0.840	n.a.	0.829	0.927
Corona_pk3	Pk	0.579	0.646	0.674	0.678	**0.705**
CPEB3_ribozyme	Pk	**0.756**	**0.756**	0.663	**0.756**	0.612
Gammaretro_CES		**0.983**	0.948	**0.983**	0.935	**0.983**
Hammerhead_1		**1.000**	0.474	0.621	0.831	0.614
Hammerhead_3		**1.000**	0.960	**1.000**	**1.000**	**1.000**
HDV_ribozyme	Pk	0.709	-0.018	**0.784**	0.388	0.396
IRES_c-myc		-0.004	0.079	0.286	-0.002	**0.350**
R2_retro_el		**1.000**	0.842	0.946	0.987	0.890
RNAIII		0.467	0.595	n.a.	0.479	**0.830**
RNase_MRP	Pk	**0.626**	0.423	0.457	0.271	0.575
rne5		**0.994**	0.969	0.975	0.762	0.923
RydC	Pk	0.466	0.562	**0.608**	0.466	-0.020
s2m		0.739	**1.000**	0.774	0.652	0.861
Telomerase-cil		**1.000**	0.937	0.921	**1.000**	0.953
Telomerase-vert	pk	**0.918**	0.751	n.a.	n.a.	0.820
Vimentin3		0.741	-0.016	0.184	**0.771**	0.629
Y		**1.000**	**1.000**	0.925	**1.000**	**1.000**

mean		0.759	0.651			0.703
mean	knetfold	0.750	0.645		0.680	0.696
mean	pfold	0.756	0.635	0.693	0.682	0.673

Performance comparisons on the RNA STRAND-Rfam dataset. We list the MCC for different alignments. Best performance indicated in **bold**, n.a. means that data is not available due to length restrictions on the respective server, pk denotes structures that contain a pseudo-knot. As there are many pseudo-knotted structures in this dataset, KNetFold was used in the "Check pseudoknot" mode. The MCCs take into account the pseudo-knots.

We also used the Rfam subset that was used to evaluate the performance of KNetFold [38]. However, we did not use the same procedure to prune alignments down to a maximum of 40 sequences. Therefore, the MCCs reported here cannot directly be compared to the ones in [38]. The MCC we achieve with the RIBOSUM variant of RNAalifold is 0.818. This is again a significant improvement over the MCC of 0.604 achieved by the 2002 variant.

When considering an almost complete set of about 570 Rfam alignments (a few alignments that for various reasons are problematic were removed), the mean MCC of RNAalifold 2002 is 0.729, the new RNAalifold with RIBOSUM matrices achieves a mean MCC of 0.790, while McC_mea achieves 0.742.

In Table 3, the performance of the new RNAalifold variants using the "other MCC" variant and the results when using Clustal realigned sequences are shown.

**Table 3:** Results using alternative MCC and alignment

Program or variant	MCC	Other MCC	Clustal MCC
RNAalifold 2002	0.831	0.814	0.708
RNAalifold new	0.845	0.819	0.711
RNAalifold RIBOSUM	**0.937**	0.871	**0.788**
RNAalifold 2002 centroid	0.828	0.815	0.693
RNAalifold new centroid	0.848	0.834	0.712
RNAalifold RIBOSUM centroid	0.934	**0.896**	0.780
Pfold	0.765	0.739	0.601
KNetFold	0.866	0.808	0.761
McC_mea	0.837	0.816	0.716

Performance comparisons on the CMfinder-SARSE dataset. We list the mean MCC for different programs. Best performance indicated in **bold**. Other MCC is the variant counting every wrongly predicted pair as false positive, Clustal MCC is the MCC as introduced by Gardner *et al*. [39] applied to alignments realigned using Clustal [40].

#### Effects on predicted structures

Over all, there are two main reasons why prediction using the RIBOSUM variant of RNAalifold will give better predictions than the 2002 variant. By treating gaps as if they were bases, the 2002 implementation sometimes assigns much too unfavorable energies to loops containing gaps in a small number of sequences. As a consequence, these loops cannot be part of the consensus structure. Examples for this effect are *GcvB*, where an interrupting bulge loop in the consensus structure actually exists in only one sequence, or the Hammerhead ribozyme, where a large insertion within a hairpin loop is present in about a third of the sequences.

The beneficial effect of using the RIBOSUM matrices is mostly due to the possibility to assign covariance boni to certain base pairs even if not much (or even no) covariation actually occurred. This makes it possible to compensate for a few contradicting base pairs, whether they are due to alignment errors or to a slightly different structure for some sequences. Predictions that benefit from that effect are e.g. the Enterovirus 5' cloverleaf, the Snake H/ACA box small nucleolar RNA or the UnaL2 LINE 3' element. A mixture of both effects is seen in the R2 RNA element as well as in the Hammerhead ribozyme. The detailed results for these molecules can be seen in the additional files 5, 6, 7, 8, 9 and 10 or in the online supplement.

### Detection of ncRNAs

AlifoldZ detects structural non-coding RNAs by comparing the energy of the native alignment to the energies of a population of randomized control alignments via a *z*-score. Here, the better predictive power of the new RIBOSUM approach directly translates into increased ability to distinguish evolutionary conserved RNAs from randomized controls. The RIBOSUM approach achieves an AUC of 0.969 compared to 0.954 for both the 2002 implementation and the new RNAalifold. The performance boost comes mainly from additional bonus energies derived from covariance scoring. In the RIBOSUM approach these energies have a much higher contribution than in the conventional model thereby favoring true conservation patterns by giving a lower total free energy and hence a lower *z*-score. This beneficial effect is not observed in the case of the SCI, where the RIBOSUM covariance energies even result in a performance drop (AUC 0.767) compared to the other two implementations (new: 0.917, 2002: 0.916). The SCI is a conservation measure that compares the consensus free energy to the mean free energy of the single sequences. The covariance energies are important for the high discrimination capability of the SCI, but with the RIBOSUM scoring model the over-emphasis of the covariance energy contributions blurs the signal for true conservation. If we neglect the covariance score for the computation of the SCI, the effect is much smaller (AUC 0.907). We expect, however, that the RIBOSUM approach will perform well on purely structure-based similarity or distance measures.

### Computational requirements

**Figure 4:**
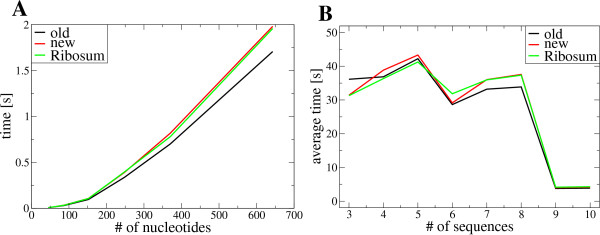
**Time series for the old, new and RIBOSUM RNAalifold variants.A**: Folding different alignments with 4 sequences and different lengths. **B**: Folding a different number of random sequences from the same alignment (1716 nt).

Theoretically, the new and the old RNAalifold variants have the same space (O(*n*^2^)) and time (O(*Nn*^3^)) complexity, with *N *sequences in an alignment of length *n*. However, neglecting possible base pairs with a conservation score below a certain cutoff (e.g. if more than 50% of the sequences cannot form a base pair) dramatically reduces computation time without affecting the results. As an example, folding a subset of five randomly chosen sequences of a ribosomal SSU alignment (length 1716 nt) takes an average of about 42.2 seconds, while using 10 sequences of the same alignment takes about 3.8 seconds on an Intel Xeon 2.8 GHz processor (Figure 4). The RIBOSUM matrices make it much harder to exclude base pairs from the outset. Thus, the RIBOSUM variant is by far the slowest option on alignments with many rather diverse sequences.

## Conclusion

We have shown here that the performance of RNAalifold can be improved to be competitive with all recently published alignment-based consensus structure prediction tools. This improvement is reached by a more accurate treatment of gaps and an elaborate model for the evaluation of sequence covariations that resembles the RIBOSUM matrices. The gain in performance is achieved at negligible extra computational cost and without dramatic changes to the implementation. While a sequence weighting scheme apparently can yield further improvements on good alignments, this makes the procedure less resilient towards mis-alignments. It seems, therefore, that the approach is essentially limited by the quality of the input alignments.

## Authors' contributions

SHB designed and implemented the new version of RNAalifold, ILH and PFS initiated the study and contributed to the theory, SW derived and calculated the RIBOSUM-like scores, ARG evaluated the performance for structured RNA detection. All authors closely collaborated in writing the manuscript.

## Availability and requirements

RNAalifold is part of the ViennaRNA software package, the new version can be downloaded for Linux as a tar archive at: .

The electronic supplement of this paper can be found at 

## Supplementary Material

Additional file 1 **Additional results. **Results of various unsuccessful approaches to increase the accuracy of RNAalifold. Click here for file

Additional file 2 **Stochastic backtracking.** Detailed description of stochastic backtracking algorithm for consensus structure prediction using RNAalifold. Click here for file

Additional file 3 **Datasets.** List of the datasets used for evaluating performance. Click here for file

Additional file 4 **Alignment and structure of SNORD86.** The Rfam alignment and reference structure of SNORD86 together with the energies of the structure on the single molecules. Click here for file

Additional file 5 **Enterovirus 5' cloverleaf structure.** Analysis of the effects leading to better prediction of the Enterovirus 5' cloverleaf structure. Click here for file

Additional file 6 **GcvB structure**. Analysis of the effects leading to better prediction of the GcvB structure. Click here for file

Additional file 7 **Snake H/ACA snoRNA structure.** Analysis of the effects leading to better prediction of the Snake H/ACA snoRNA structure. Click here for file

Additional file 8 **Hammerhead Rybozyme structure.** Analysis of the effects leading to better prediction of the Hammerhead Rybozyme structure. Click here for file

Additional file 9 **R2 RNA element structure.** Analysis of the effects leading to better prediction of the R2 RNA element structure. Click here for file

Additional file 10 **UnaL2 LINE 3' element structure.** Analysis of the effects leading to better prediction of the UnaL2 LINE 3' element structure. Click here for file
