# Supplementary material for: RNAalifold: improved consensus structure prediction for RNA alignments
Source: BMC Bioinformatics. 2008 Nov 11;9:474. doi: 10.1186/1471-2105-9-474 (PMC2621365; doi:10.1186/1471-2105-9-474)
Supplement: Additional file 1 — Additional results. Results of various unsuccessful approaches to increase the accuracy of RNAalifold. [file 1471-2105-9-474-S1.pdf]

Computation type	MCC
RIBOSUM best	0.936
New best	0.916
2002 (Old) best	0.884
RIBOSUM	0.878
Pfold-like best	0.876
New	0.845
2002	0.831
weighted	0.828
Pfold-like	0.760
RIBOSUM using Andronescu parameters	0.710
Andronescu parameters	0.674

Mathews correlation coefficient (MCC) of different ways to score the conservation term in RNAalifold, on the CMfinder SARSE data-set.
Best (if applicable) is the best performance achieved, the other uses the (old) default parameters.
All computations (except the 2002 variant) were done using the new variant of not counting gaps as bases for energy computations.

Computation type	Dataset	MCC Turner	MCC Andronescu
RIBOSUM	CMfinder	0.936	0.904
New	CMfinder	0.845	0.832
2002	CMfinder	0.831	0.819
RIBOSUM	RNASTRAND-Rfam	0.759	0.758
New	RNASTRAND-Rfam	0.710	0.688
RIBOSUM	Rfam	0.790	0.766
New	Rfam	0.746	0.727
2002	Rfam	0.729	0.718

Mean results when using the parameters of Andronescu et al. versus the usual Turner parameters. While some predictions are better when Andronescu's parameters are used, on average they do slightly worse than the Turner parameters.
