# Supplementary material for: RNAalifold: improved consensus structure prediction for RNA alignments
Source: BMC Bioinformatics. 2008 Nov 11;9:474. doi: 10.1186/1471-2105-9-474 (PMC2621365; doi:10.1186/1471-2105-9-474)
Supplement: Additional file 2 — Stochastic backtracking. Detailed description of stochastic backtracking algorithm for consensus structure prediction using RNAalifold. [file 1471-2105-9-474-S2.pdf]

1 Stochastic Backtrack of RNA consensus structures

For the stochastic backtracking of `RNAalifold`, we need the probabilities for the single building blocks of secondary structures. These probabilities can easily be computed out of the matrices of the partition function version of `RNAalifold`:

$Q(i,j)$ The partition function of the sequence stretch $[i,j]$

$Q^B(i,j)$ The partition function of the sequence stretch $[i,j]$ given that i,j is a base pair.

$Q^M(i,j)$ The partition function of the sequence stretch $[i,j]$ as a part of a multi loop containing at least one component.

$Q^{M1}(i,j)$ The partition function of the sequence stretch $[i,j]$ as a part of a multi loop containing exactly one component starting at i.

Starting with the unpaired chain, we look for the first base that is paired:

$$p_{\text{i is paired}} = 1 - \frac{Q(i+1,n)}{Q(i,n)} \tag{1}$$

When an i is found, the respective pairing partner j is sought:

$$p_{\text{pair}}(j|i) = \frac{\exp\left(\frac{-\beta\gamma(i,j)}{kT}\right) Q^B(i,j)Q(j+1,n)}{Q(i,n)} \tag{2}$$

Having found a base pair, we have to find out whether it closes a hairpin loop, interior loop or multi loop (the interval $[j+1,n]$ is backtracked later on):

$$\begin{aligned}
p_{\text{hairpin}}(i,j) &= \frac{\exp\left(\frac{-\sum_\alpha \mathfrak{H}(i,j,\alpha)}{kT}\right)}{Q^B(i,j)} \\
p_{\text{interior loop }k,l}(i,j) &= \frac{\exp\left(\frac{-\sum_\alpha \mathfrak{I}(ij,kl,\alpha)}{kT}\right) Q^B(k,l)\exp\left(\frac{-\beta\gamma(k,l)}{kT}\right)}{Q^B(i,j)} \\
p_{\text{multi loop }u}(i,j) &= \frac{Q^M(i+1,u)Q^{M1}(u+1,j-1)\exp\left(\frac{-\mathfrak{a}}{kT}\right)}{Q^B(i,j)}
\end{aligned}$$

In case a hairpin loop is found, backtracking stops for this interval, in case of an interior loop, the next base pair k,l is backtracked. When a Multi loop is found, we have to backtrack $Q^M(i+1,u)$ and $Q^{M1}(u+1,j-1)$. Q^M backtrack works just like backtracking Q:

$$\begin{aligned}
p_{\text{i is paired in }Q^M(i,u)} &= \frac{Q^M(i+1,u)}{Q^M(i,u)} \\
p_{\text{pair}}(j|i,Q^M(i,u)) &= \frac{\exp\left(\frac{-\beta\gamma(i,j)}{kT}\right) Q^B(i,j)\left(\frac{-\beta\gamma(i,j)}{kT}\right)\exp\left(\frac{-\mathfrak{b}}{kT}\right)}{Q^M(i,u)} * \\
&* \quad \frac{Q^M(j+1,u)+\exp\left((u-j)\frac{-\mathfrak{c}}{kT}\right)}{Q^M(i,u)}
\end{aligned}$$

When $Q^M(j+1,u)=0$, backtrack stops. The probability for j in $Q^{M1}(i,v)$ is:

$$p(j|Q^{M1}(i,v) = \frac{Q^B(i,j)\exp\left(\frac{-\beta\gamma(i,j)}{kT}\right)\exp\left(\frac{-\mathfrak{c}(v-j)}{kT}\right)}{Q^{M1}(i,v)} \tag{3}$$

In this computation, $\mathfrak{H}(i,j,\alpha)$ and $\mathfrak{I}(ij,kl,\alpha)$ are the hairpin and interior loop energies for the sequence α of the alignment, respectively. $\mathfrak{a},\mathfrak{b},\mathfrak{c}$ are the multi loop energy terms for closing a multi loop, a stem within a multi loop and a free base within a multi loop, resp. $\gamma(i,j)$ is the covariation penalty and β its weighting factor, k is Boltzmann's constant and T the absolute temperature.
