# Supplementary material for: RNAalifold: improved consensus structure prediction for RNA alignments
Source: BMC Bioinformatics. 2008 Nov 11;9:474. doi: 10.1186/1471-2105-9-474 (PMC2621365; doi:10.1186/1471-2105-9-474)
Supplement: Additional file 3 — Datasets. List of the datasets used for evaluating performance. [file 1471-2105-9-474-S3.pdf]

Alignments and structures used to assess the function of RNAalifold

Datasets used for assessing predictive power or the new RNAalifold. If the reference structure is a prediction, information of what program was used is included. If only a name is given, information of how prediction was done is not available. CM, KN and ST denote presence of structure/alignment pair also in CM_finder, KNetFold or RNA STRAND dataset, respectively. pk means that the reference structure contains a pseudo-knot.

0.1 CMfinder dataset

name	pk	also in other dataset	predicted
Antizyme_FSE	pk		
ctRNA_pGA1			
Entero_5_CRE		KN	
Entero_CRE		KN	
GcvB			predicted by A. Bateman
glmS			
HACA_sno_Snake		KN	predicted with pfold
HCV_SLIV		KN	predicted with ILM
HDV_ribozyme	pk	ST	
HepC_CRE			
Histone3		KN	
Hsp90_CRE			
IBV_D-RNA			
Intron_gpII			predicted with pfold
IRE KN			
let-7			predicted with pfold
lin-4			predicted with pfold
Lysine			
mir-10			predicted with pfold
mir-194			predicted with pfold
mir-BART1			
nos_TCE			
Purine		KN	
Rhino_CRE			
RNA-OUT			predicted with pfold
rncO			
Rota_CRE			predicted with pfold
s2m		ST KN	
SCARNA14 predicted with RNAalifold			
SCARNA15 predicted with RNAfold			
SECIS		KN	
SNORA14			predicted with RNAfold
SNORA18			predicted with RNAfold
SNORA38			predicted with RNAfold
SNORA40			predicted with RNAalifold
SNORA56			predicted with RNAfold
SNORD105			predicted with RNAalifold
SNORD64			predicted with RNAalifold
SNORD86			predicted with RNAalifold
snoU83B			predicted with RNAalifold
TCV_H5			
TCV_Pr			
Tymo_tRNA-like	pk	KN	
ykoK			predicted by J. E. Barrick and R. R. Breaker

KNetFold dataset

name	pk	also in other dataset	predicted
5S			
ctRNA_pND324			
Entero_5_CRE		CM	
Entero_CRE		CM	
Entero_OriR	pk		predicted with pfold
Gammaretro_CES			
HACA_sno_Snake		CM	predicted with pfold
Hammerhead_1		ST	
Hammerhead_3		ST	
HCV_SLIV		CM	predicted with ILM
HCV_SLVII			predicted with RNAalifold
HCV_X3			
HepC_CRE			
HgcC			
Histone3		CM	
HIV_FE			
HIV_GSL3			predicted with pfold
HIV_PBS			predicted with pfold
IRE CM			
JEV_hairpin			
K_chan_RES			
mir-395			predicted with pfold
Purine CM			
Retro_dr1			
Retroviral_psi			
RSV_PBS			
s2m		ST CM	
satBaMV_CRE			
SECIS		CM	
SL1			
SL2			
snoPyro_CD			predicted with pfold
snoR71			predicted with pfold
SNORA73			
SNORD113			
SNORD115			predicted with pfold
SNORD36			predicted with pfold
snosnR60_Z15			predicted by S. Griffiths-Jones
SRP_bact			
TAR			
Thr_leader			
Tombus_3_III			
tRNA			
Tymo_tRNA-like	pk	CM	
U4			
U7			predicted with pfold
UnaL2			
VA			

RNA STRAND-Rfam dataset

name	pk	also in other dataset	predicted
7SK			
bicoid_3			
Corona_pk3	pk		
CPEB3_ribozyme	pk		
Gammaretro_CES			
Hammerhead_1		KN	
Hammerhead_3		KN	
HDV_ribozyme	pk	CM	
IRES_c-myc			
R2_retro_el			
RNAIII			
RNase_MRP	pk		
rne5			
RydC	pk		
s2m		CM KN	
Telomerase-cil			
Telomerase-vert	pk		
Vimentin3			
Y			
