# Supplementary material for: RNAalifold: improved consensus structure prediction for RNA alignments
Source: BMC Bioinformatics. 2008 Nov 11;9:474. doi: 10.1186/1471-2105-9-474 (PMC2621365; doi:10.1186/1471-2105-9-474)
Supplement: Additional file 4 — Alignment and structure of SNORD86. The Rfam alignment and reference structure of SNORD86 together with the energies of the structure on the single molecules. [file 1471-2105-9-474-S4.pdf]

```
 Ref:<<<<<<...<..<<.....<<<<<<<<<<<.<.<<<<...<<<<<....>>>.>>.>>>>.>.>>..>>>>>>>>>>>>...>>>>>>     E
AC15 AUUUCAUCAG--AGAACACAAAAGAACAUUCUUAAAACUAAAUAGCUCUGCUAUGGGGGUGAUGGCCAGUCCUGGUGUCUGAGUGAUU  20.10
AC12 UGCCUUUCAG--A----AGAAGAGGCGGACCUGACCUAUACAGGCCUCUGCUAUGGGGAUGAUGGCCAGUCCUGGUGUCUGAGUGAUU   4.20
AL66 GAUAUUUAAGUCAUUUAACAUAUUUUGUUCUGGACCUAUACAGGCCUCUGCUAUGGGGGUGAUGUUCAGUCCUGGUGUCUGAGUGAUU  10.40
AL83 GAUUACAAUG--AUGGCUGACCAGGGCAACCUGACCUAUACAGGCCUCUGCUAUGGGGGUGAUGGCCAGUCCUGGUGUCUGAGUGAUU -24.30
AJ31 GAUCACGGUG--AUGGCUGACCAGGGCUCCCUGACCUAUACAGGCCUCUGCUAUGGGGGUGAUGGCCAGUCCUGGUGUCUGAGUGAUU -31.60
AY00 GAUCACAGUG--AUGGUUGACCAGGGCUCCCUGACCUAUACAGGCCUCUGCUAUGGGGGUGAUGGCCAGUCCUGGUGUCUGAGUGAUU -30.50
```

Rfam alignment and reference secondary structure of the SNORD86 RNA. As can be seen, the top 3 sequences will have positive energies if forced in the reference secondary structure. Thus, the alignment is clearly not right for these three sequences.
