# Supplementary material for: RNAalifold: improved consensus structure prediction for RNA alignments
Source: BMC Bioinformatics. 2008 Nov 11;9:474. doi: 10.1186/1471-2105-9-474 (PMC2621365; doi:10.1186/1471-2105-9-474)
Supplement: Additional file 5 — Enterovirus 5' cloverleaf structure. Analysis of the effects leading to better prediction of the Enterovirus 5' cloverleaf structure. [file 1471-2105-9-474-S5.pdf]

Entero_5_CRE

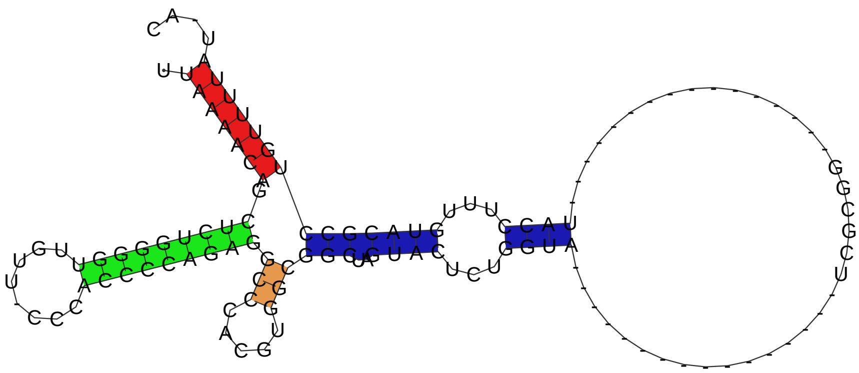

Rfam reference structure of Entero_5_CRE

Positive effect of the RIBOSUM covariance scoring on predictive power – Entero_5_CRE: The closing stem of the Multi loop (red) can not be predicted using hamming distance scoring. About 15 sequences of an alignment of 160 can not form it.

The alignment shows a representative sample of the 160 sequences in the `Rfam`. The third sequence of the picture is one of the examples where the closing stem can not be formed. Because there is little compensatory mutation, the covariance score is negative in the original `RNAalifold`.

When the new energy parameters are used, there is a – small – positive effect of not having any mutations, also. In this case, the huge number (more than 140 or about 90%) of sequences that contain no mutations is enough to outweigh the 10 structures that can not form the structure.

```
                              ******  **** **    ******   *** *  ** **  * *****  *******  * ** **********
  L76402.1/1-91 UUAAAACAGCUCUGGGGUUGUUCCCACCCCAGAGGCCCACGUGGCGGCUAGUACUCUGGUAUCGCGGUACCUUUGUACGCCUGUUUUAUAC
  D90457-1/1-91 UUAAAACAGCUCUGGGGUUGUUCCCACCCCAGAGGCCCACGUGGCGGCCAGUACACUGGUAUCACGGUACCUUUGUACGCCUGUUUUAAAC
AJ577593-1/1-78 GU------------GGGUUGUACCCAUCCACAGGGCCCACUGGGC-GCCAGCACUCUGGUAUUGCGGUACCUUAGUGCGCCUGUUUUAUAU
AF448782-1/1-91 UUAAAACAGCUCUGGGGUUGUUCCCACCCCAGAGGCCCACGUGGCGGCUAGUACACUGGUAUUACGGUACCCUUGUACGCCUGUUUUAUAC
AF465516-1/1-90 UUAAAACAGCCUGUGGGUUGAUCCCACCCACAGGGCCCAUUGGGC-GUCAGCACCCUGGUAUCACGGUACCUUUGUGCGCCUGUUUUAUAU
  L76406-1/1-91 NNAAAACAGCUCUGGGGUUGUUCCCACCCCAGAGGCCCACGUGGCGGCUAGUACUCCGGUAUUACGGUACCCUUGUACGCCUGUUUUAUAC
  X80059-1/1-90 UUAAAACAGCCUGUGGGUUGUACCCACCCACAGGGCCCACUGGGC-GCUAGCACACUGGUAUCACGGUACCUUUGUGCGCCUGUUUUAUAC
  X84981-1/1-90 UUAAAACAGCCUGUGGGUUGUACCCACCCACAGGGCCCACUGGGC-GCCAGCACUCUGGUAUCGCGGUACCUUUGUGCGCCUGUUUUGUUU
AJ133661-1/1-90 UUAAAACAGCCUGUGGGUUGUUCCCACCCACAGGGCCCACUGGGC-GCCAGCACACUGGUAUCACGGUACCUUUGUGCGCCUGUUUUAUCC
  D00435-1/1-90 UUAAAACAGCCUGUGGGUUGUUCCCACCCACAGGGCCCACUGGGC-GCUAGUACACUGGUAUCACGGUACCUUUGUGCGCCUGUUUGACUU

      structure .<<<<<<<.(((((((((.......)))))))))(((.....)))(((..((((...((((......))))...)))))))>>>>>>>...
```

Alignment and dot bracket reference structure of Entero_5_CRE.
