# Supplementary material for: RNAalifold: improved consensus structure prediction for RNA alignments
Source: BMC Bioinformatics. 2008 Nov 11;9:474. doi: 10.1186/1471-2105-9-474 (PMC2621365; doi:10.1186/1471-2105-9-474)
Supplement: Additional file 6 — GcvB structure. Analysis of the effects leading to better prediction of the GcvB structure. [file 1471-2105-9-474-S6.pdf]

Alignment and structure of GcvB.

This is a part of the 5' end of the `Rfam` alignment GcvB. The inner (green) stem usually has a single bulge loop. However, due to the one sequence that has a 3nt insertion (yellow column), the original `RNAalifold` treats all sequences as if they had a bulge loop there. Because of this, it prefers the large interior loop over two small bulges, which leads to the 3 missing base pairs (magenta). Treating gaps as not being there – instead of as "phantom" nucleotides – remedies that.

```
  U00096-2/2940718-2940864 ACUUC-CUGAGCCGGAACGAAAAGUUUUAUCGGAAUGCGU-GUUCUGGUGAA---CUUUUGGCUUACG--GU
     U29581-1/24251-24397 ACUUC-CUGAGCCGGAACGAAAAGUUUUAUCGGAAUGCGU-GUUCUGGUGAA---CUUUUGGCUUACG--GU
          U01030-1/175-29 ACUUC-CUGAGCCGGAACGAAAAGUUUUAUCGGAAUGCGU-GUUCUGGUGAA---CUUUUGGCUUACG--GU
  AE016765-1/195503-195649 ACUUC-CUGAGCCGGAACGAAAAGUUUUAUCGGAAUGCGU-GUUCUGGUGAA---CUUUUGGCUUACG--GU
BX950851-1/1137282-1137144 ACUUC-CUGGGCCGGAACGAAAAGUGCGGAGGGG------UGACCUGAGGUG---CUUUUGGCUUGUG--GU
    BX571861-1/50378-50242 ACUUC-CUAAGCCGGAACGAAAAGUGA-AUCAGCUUA----ACGCUAUGAAA---CUUUUGGCUUUGUG-GU
    AL627277-1/14855-15001 ACUUC-CUGAGCCGGAACGAAAAGUUUUAUCGGAAUGCGU-GUUCUGAUGGG---CUUUUGGCUUACG--GU
    AE008836-1/18189-18335 ACUUC-CUGAGCCGGAACGAAAAGUUUUAUCGGAAUGCGU-GUUCUGAUGGG---CUUUUGGCUUACG--GU
  AE016843-1/272852-272998 ACUUC-CUGAGCCGGAACGAAAAGUUUUAUCGGAAUGCGU-GUUCUGAUGGG---CUUUUGGCUUACG--GU
  AE016987-1/274185-274331 ACUUC-CUGAGCCGGAACGAAAAGUUUUAUCGGAAUGCGU-AUUCUGGUGAA---CUUUUGGCUUACG--GU
      AE013916-1/1147-1288 ACUUCCCCUAGCCGGAACGAAAAGUAGGUUUGGUAUCCCAGGUACUGAAAUG---CUUUUGGCUUUGUG-GU
    AJ414146-1/50795-50654 ACUUCCCCUAGCCGGAACGAAAAGUAGGUUUGGUAUCCCAGGUACUGAAAUG---CUUUUGGCUUUGUG-GU
  AE017137-1/228019-228160 ACUUCCCCUAGCCGGAACGAAAAGUAGGUUUGGUAUCCCAGGUACUGAAAUG---CUUUUGGCUUUGUG-GU
          U32799-1/164-25 UCCUU-ACAAUACAACCAUACUUAAUG-AUUGGUAAUUCC-UUACUAGUUAA---UGAGUUUCAGACUUAAG
      AE006092-1/5387-5242 CCCUCUAAAAUGCGCGCAUACUUAAUG-AUUGGUAAUUCC-UUACUGGUUAAGAGUUUCGGACUUAAG----
      AE004173-1/8619-8498 CGCAACGGCGGCCUGAACGGCUUGAUU-UCCU-------------UGUAAAU---CAAUAGGCUUAGUU-GU
  AE016797-1/298902-299024 CGCAACGCUAGCCUGAACGGCUUGAUU-UCUU-------------UGUAAAU---CAAUUGGCUUAGUU-GU

            GcvB reference .........((((..((....((((((.((((((((.....)))))))))))...))))))))).........
                Originale .........(((((......((((....((((((.........))))))......))))))))).........
                      New .........(((((......(((((((.((((((.........)))))))))...))))))))).........
```
