# Supplementary material for: RNAalifold: improved consensus structure prediction for RNA alignments
Source: BMC Bioinformatics. 2008 Nov 11;9:474. doi: 10.1186/1471-2105-9-474 (PMC2621365; doi:10.1186/1471-2105-9-474)

Structure of HACA_sno_Snake

Comparison of the reference (Rfam) structure of HACA_sno_Snake and predicted structures. The second stem of the snoRNA is predicted only if the covariance terms are downweighted. However, the more sophisticated RIBOSUM scoring scheme will allow the correct prediction while the relative portion of the covariance in the total score is much bigger than in the original `RNAalifold`. Using the suggested new parameters ($\beta = 0.6$ and $\delta = 0.5$ in the RIBOSUM case) is sufficient to predict both stems.

Reference Structure of HACA_sno_Snake

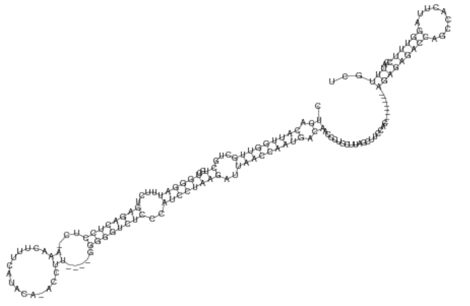

Structure using the original `RNAalifold`

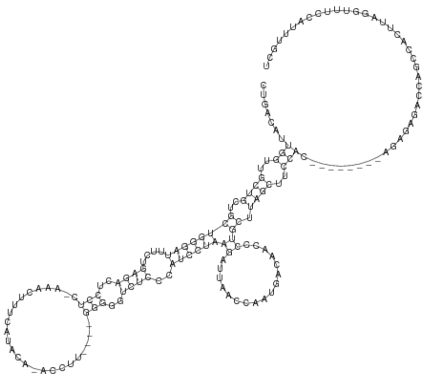

Structure using RIBOSUM

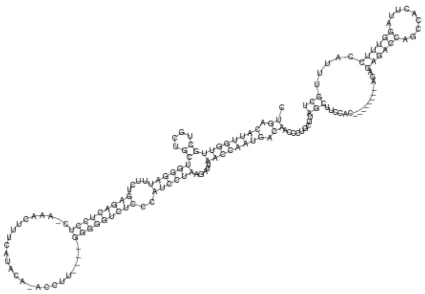

Supplement: Additional file 7 — Snake H/ACA snoRNA structure. Analysis of the effects leading to better prediction of the Snake H/ACA snoRNA structure. [file 1471-2105-9-474-S7.pdf]
