# Supplementary material for: RNAalifold: improved consensus structure prediction for RNA alignments
Source: BMC Bioinformatics. 2008 Nov 11;9:474. doi: 10.1186/1471-2105-9-474 (PMC2621365; doi:10.1186/1471-2105-9-474)

Hammerhead 1 Ribozyme

Comparison of the reference Rfam structure of the Hammerhead1 Ribozyme and predicted structures. Two of the stems can not be predicted using the original `RNAalifold`. Better treatment of gaps is enough to predict all stems, but only using the RIBOSUM scoring scheme predicts all base pairs correctly.

Reference structure

Identical to RIBOSUM structure

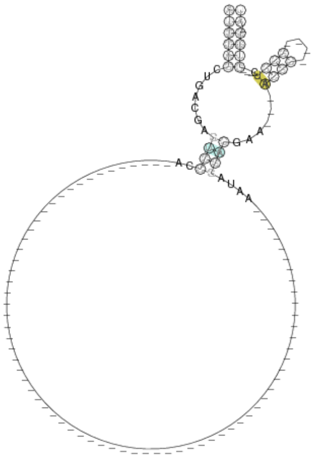

Structure using the original `RNAalifold`

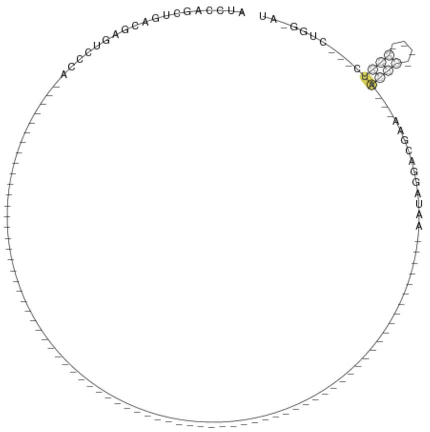

Structure when using the new gap treatment

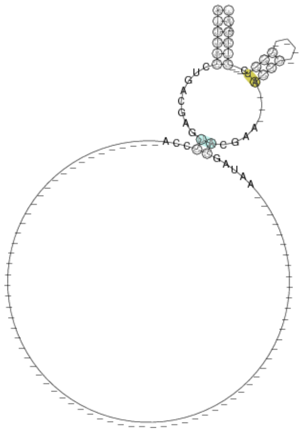

When looking at the structure annotated alignment, one can see that the blue hairpin loop is much too long if the old `RNAalifold` is used, and therefore will not be formed. This in turn leads to a very long, energetically highly unfavorable interior loop between the green and the red stem. Thus, the red stem can also only be correctly predicted if gaps are not used to evaluate loop energies.

The two outer blue base pairs have so many counter examples that only the RIBOSUM way of giving a small bonus to identical base pairs also leads to their inclusion into the structure.

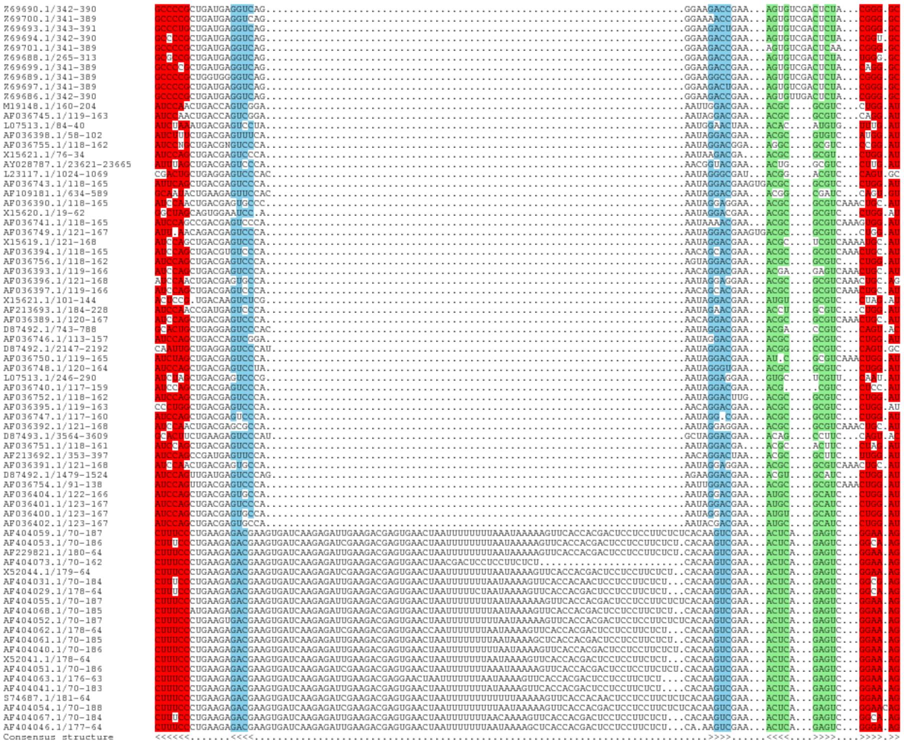

Supplement: Additional file 8 — Hammerhead Rybozyme structure. Analysis of the effects leading to better prediction of the Hammerhead Rybozyme structure. [file 1471-2105-9-474-S8.pdf]
