# Supplementary material for: RNAalifold: improved consensus structure prediction for RNA alignments
Source: BMC Bioinformatics. 2008 Nov 11;9:474. doi: 10.1186/1471-2105-9-474 (PMC2621365; doi:10.1186/1471-2105-9-474)
Supplement: Additional file 9 — R2 RNA element structure. Analysis of the effects leading to better prediction of the R2 RNA element structure. [file 1471-2105-9-474-S9.pdf]

R2 retro element

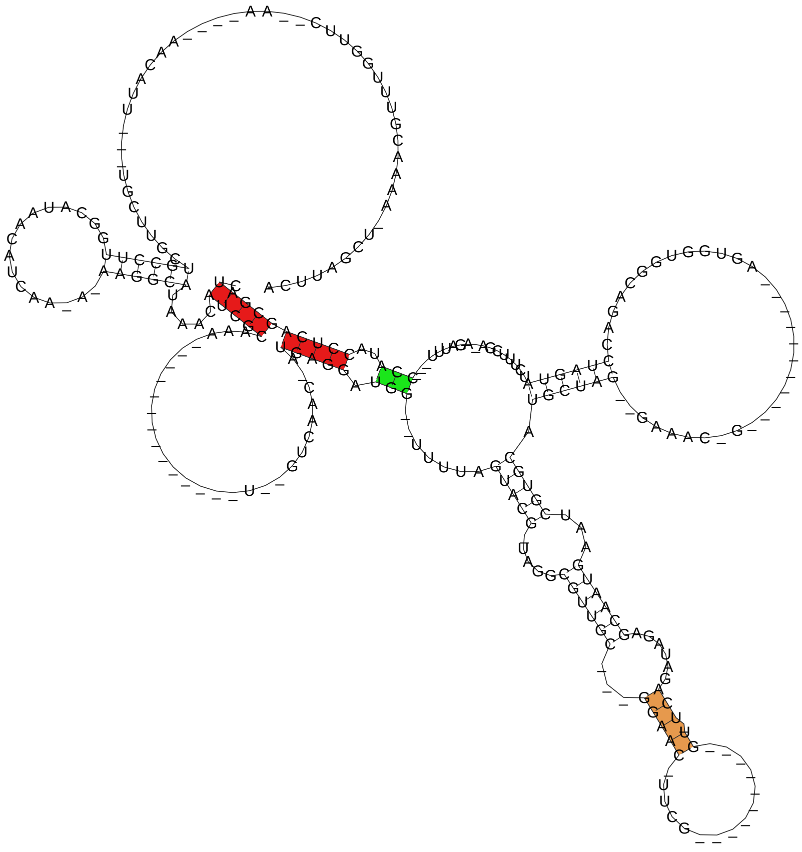

Reference secondary structure (Rfam)

While the RIBOSUM `RNAalifold` variant correctly predicts all base pairs, the colored stems can not be predicted using the original `RNAalifold` implementation.

Using the more sophisticated gap treatment, is sufficient to predict the orange and red stems. In the orange stem, the single bulge that is actually only there in one sequence, would destabilize the short stem (see alignment below). For the red stem, the size of the interior loop is much smaller in most sequences than what the old`RNAalifold` implementation uses in the energy evaluations. As the destabilizing effect of interior loops rises with their length, this leads to neglecting this stem loop.

The green stem is a good example for the superiority of the RIBOSUM scoring over the old ad-hoc scoring. Only some (20%) of the structures can not realize it, and there are some compensatory mutations: there are 3 different stems of length three, which are distributed very unevenly: one only occurs once, one twice. That will lead to quite small covariance score when using ad-hoc scoring. RIBOSUM scoring also uses small bonuses for keeping a sequence, so the

covariance term is bigger and sufficient for a correct prediction.

```
U13032.1/1423-1651 ACUUAGCU-AAAACGUUUGGUUC--AA----AACAUU---UGCUUGCUGUCUUGGCAUAACAUCAAUA-AAGGCAUAAACAUCGCAAAUAAU
  U81815-1/525-734 AUGUACUA-ACCUCUAGCUUUUCUUAU----ACCUUU---UGCCUGCUACCUUGGCAUUACAUCUAAA-AAGGUACAAACAUCGCAUU----
U13031-1/1423-1644 ACUUAGCU-AAAUCGUUUGGUUC--AA----AACAUU---UGCUUGCUGUCUUGGCAUAACAUCAAUA-AAGGCAUAAACAUCGCAAAUAAU
U13033-1/1423-1658 ACUUAGCU-AAAACGUUUGGUUC--AA----AACAUU---UGCUUGCUGUCUUGGCAUAACAUCAAUA-AAGGCAUAAACAUCGCAAAACAA
  U81811-1/530-751 ACGUAGCUGAGCGCGUUUGAUAUA-GU----GAUAAA---UGCCUGCUGCCUGGGCUCAACAUCAAAUGCAGGCAUAAACAUCGCAAC----
  U81813-1/515-708 ACUUAGCC-UAUACACUAUGUUGG-AG----AGAGAC----GCUUGCUACCUAGGCAUAAUGUGAA-U-UAGGUAUAAACAUCGUGGU----
  U81985-1/508-709 ACCUAGCC-GAAACACUAUGUUGG-AA----AGAAGA---CGCUUGCUACCUAGGCAUAAUGUAAAAU-UAGGUAUAAACAUCGCAGU----
X51967-1/3349-3585 ACUUAGCU-AAAUCGUUUGGUUC--AA----AACAUU---UGCUUGCUGUCUUGGCAUAACAUCAAUA-AAGGCAUAAACAUCGCAAAAUAA
  U81814-1/504-719 GCUUGAUC-UCUGUAUUUGUUUCU-AUUUUGAACAUU---UGCCUGCUACCUUGGCAUAACAUCAA-U-AAGGUACAAACAUCGCAAA----
  U81812-1/515-708 ACUUAGCC-UAUACACUAUGUUGG-AG----AGAGAC----GCUUGCUACCUAGGCAUAAUGUGAA-U-UAGGUAUAAACAUCGUGGU----
  U81809-1/517-738 ACUUAAUC-A-UGCACCACGAAC--AU----AUUGAUGAAAGCUUGCUGCCGUAGCAUAACACCUAAAAACGGCAUAAAAUUCGCAU-----
U13035-1/1423-1647 ACGUAGCUUAAAACGUUUGGUUCA-CA----UACAUC---UGCCUGCUGCCUUGGCACAAUAUCAA-A-AAGGCAUAAACAUCGCACAUAAU
  U81810-1/484-703 ACUUAAUC-A-UGCAUCACGUUC--AU----AUUAAA---AGCUUGCUGCCGUAGCAUAACAUCUAAAAACGGCAUAAAAUUCGCUUA----
U13034-1/1423-1648 ACGUAGCU-UAAACGUUUGGUUCA-CA----UACAUC---UGCCUGCUGCCUUGGCAUAAUAUCAA-A-AAGGCAUAAACAUCGCACAAUAA
AF015819-1/3308-3490 AAAUCGCA-U-UGGGAUAGGAUG--AU----AGCG------------CACCUGGUCAUCGUCUCUC-U-CAGCUGCUCA-CUUGCUGUUC--

         structure ...............................................((((((................)))))).....(((((.......

U13032.1/1423-1651 GGUAAUAUA-UAAAUUGGCUAUGAGGAUGG--UUUUAGUACG-UAGGCGUUGC---GGAAC-UUCG----------GU-UCAGAUAGAGCAA
  U81815-1/525-734 -------------GUCAUA-AAGAGG-UGG--UUUUAGUACG-UAGGCGCUGU---GGGAC-UUCA--------UUGU-CCCGGUGAUGCAG
U13031-1/1423-1644 GGUAAUAUA-UAAAUGGCU-AUGAGGAUGG--UUUUAGUACG-UAGGCGUUGC---GGAAC-UUCG----------GU-UCA------GCAA
U13033-1/1423-1658 UGGUUAUAUAUAAAUGGCU-AUGAGGAUGG--UUUUAGUACG-UAGGCGUUGC---GGAAC-UUCG----------GU-UCAGAUAGAGCAA
  U81811-1/530-751 ----------UAGCA-ACA-AGGAGGAUGGU-UUUUAGUACG-UAGGCAUUGC---GGAACCCUCAACGUGAAGAAGU-UCAGAUAGAGCAA
  U81813-1/515-708 ----------U--GUAAAC-UUGAG-UGGG--UUUUAGUACGGUAUGCGUG-----AUUAC-UUC-----------GU-AAU--------CA
  U81985-1/508-709 ----------U--GUAAAC-UUGAGG-UGG---UUUAGUACG-UAGGCGUGAU---GAUGA-CUUG----------UU-GAAG-UGAAACCA
X51967-1/3349-3585 UGGUUAUAAUUAAAUGGCU-AUGAGGAUGG--UUUUAGUACG-UAGGCGUUGC---GGAAC-UUCG----------GU-UCAUAUAGAGCAA
  U81814-1/504-719 -----------AAGUCAUC-AUAAGGUGGG--UUUUAGUACG-UAGGCGCUGU---AGAAC-UUAA--------UUGU-UCUGAUAGAGCAG
  U81812-1/515-708 ----------U--GUAAAC-UUGAG-UGGG--UUUUAGUACGGUAUGCGUG-----AUUAC-UUC-----------GU-AAU--------CA
  U81809-1/517-738 ----------------AAA-ACGAGGCGGU--UUUUAGUACG-UAGGCAUCUCUGGGGGGA-GGAU----------AU-CCACCCCAA-UGA
U13035-1/1423-1647 GGUUAU----U--UACGGCUAUGAGGAUGG--UUUUAGUACG-UAGGCGUUGC---GGAAC-UUCG----------GU-UCGGAUAGAGCAA
  U81810-1/484-703 ----------------AAA-ACGAGGCCUU--UUUUAGUACG-UAGGCGUCUUCU-GGGUC-GUAU----------GA-ACCCACA---AGA
U13034-1/1423-1648 UGGU------UAAUACGGCUAUGAGGAUGG--UUUUAGUACG-UAGGCGUUGC---GGAAC-UUCG----------GU-UCAGAUAGAGCAA
AF015819-1/3308-3490 --------UAAGUGAUAAUACCGUUG---UUUUUUUAGUGGG-UAUUCUUUU-----ACGC-UUUC----------GUAG---------GAG

         structure .....................(((((.(((.......(((((.....((((((...(((((...............)).)))......))))

U13032.1/1423-1651 UGAAUCGCGCAUGCUAG--GAAACUGA----------AGUGUUGACAGACCUAGUAUCUUUCGAUAGAUUU---CCAUACCUCCGCGAUC
  U81815-1/525-734 UGAAUCGUGACUACGAGAUUGUCC-------------AGUAGUUGGU--GCUCGUAUCUUUAGA-AGAUUU---CCUU-CCUCGGCGAUC
U13031-1/1423-1644 UGAAUCGUGCAUGCUAG--GAAACUGA----------AGUGUUGACAGACCUAGUAUCUUUCGAUAGAUUU---CCAUACCUCCGCGAUC
U13033-1/1423-1658 UGAAUCGUGCAUGCUAG-GAAAACUGACCACACGC--AGU--UGGCAGCCCUAGUAUCUUUCGAUAGAUUU---CCAUACCUCCGCGAUC
  U81811-1/530-751 UGAAUCGUGCAUGCUAG-AGUCAU---------------UGGUUCGA--CCUAGUAUCUUUCGA-AGAUUU---CCAUUCCUUCGCGAUC
  U81813-1/515-708 UGAAUCGUGCAUGCUAG-UGGGGU--------------UUGGCCUCU--ACUAGUAUCUUU-GA-AGAUUU---CCCUUCCUCAGCGAUC
  U81985-1/508-709 CGAAUCGUGCUCGCUAU---UA-C--------------GUUGGCCCUUA-AUAGUAUCUAU-GA-AGAUUU--CCCAU-CCUCAGCGGUC
X51967-1/3349-3585 UGAAUCGUGCAUGCUAG-GAAAACUGACCACACAC--AGUGUUGGCAGACCUAGUAUCUUUCGA-AGAUUU---CCAUACCUCCGCGAUC
  U81814-1/504-719 CGAGUCGUGCAUGCUAG-UCUAAC-------------AUUUCUUGCU-ACCUAGUAUCUUUAGA-AGAUUU---CCCUCCCUUAGCGGUC
  U81812-1/515-708 UGAAUCGUGCAUGCUAG-UGGGGU---------------UUG-GCCUCCACUAGUAUCUUU-GA-AGAUUU---UCCUUCCUCAGCGAUC
  U81809-1/517-738 UGAAUCGUGCAUGCACACUCACAUAGGCCAAACAAGAACUACCGGCCGAGUGUGUAUCUUUUGA--GAUUU---UACGACCUUAGCGA-C
U13035-1/1423-1647 UGAAUCGUGCAUGCUAG---GAACUGACC--------AAAUAACGCAGCCCUAGUAUCUUUCGA-AGAUUU---CCAUACCUUUGCGAUC
  U81810-1/484-703 UGAAUCGUGCAUGCACACUCUUAUUGGCUAAAUAAC-AUCAUAACCUGAGUGUGUAUCUUUUGA-AGAUUUUUACGAU-CCUUAGCGAAC
U13034-1/1423-1648 UGAAUCGUGCAUGCUAG--GAAACUGACCA-------AAUGGUGGCAGCCCUAGUAUCUUUCGA-AGAUUU---CCAUACCUUUGCGAUC
AF015819-1/3308-3490 CGAGUCCCACACUCU-U--GGAGC-------------AAUCCGGG-----GUAGUGCCUAA--A-CGCAUU---UCUU-CAAC-GUAAAA

         structure ))...))))).((((((.................................))))))..................)))..)))))))))).
```
