# Supplementary material for: RNAalifold: improved consensus structure prediction for RNA alignments
Source: BMC Bioinformatics. 2008 Nov 11;9:474. doi: 10.1186/1471-2105-9-474 (PMC2621365; doi:10.1186/1471-2105-9-474)

UnaL2 LINE 3' element

Comparison of the NMR derived reference Rfam structure of the UnaL2 LINE 3' element and predicted structures. Using the original hamming distance covariance scoring of `RNAalifold`, the stem is not correctly predicted.

Reference Structure of UnaL2 LINE 3' element

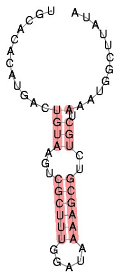

Reference Alignment of UnaL2 LINE 3' element

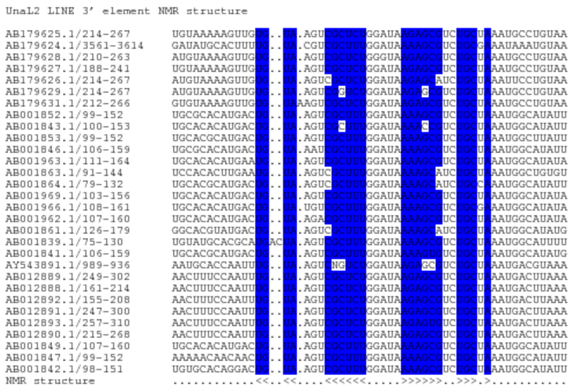

Structure using the original `RNAalifold`

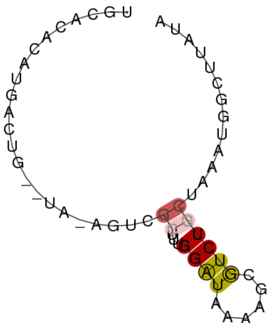

Alignment of UnaL2 LINE 3' element, original `RNAalifold` structure

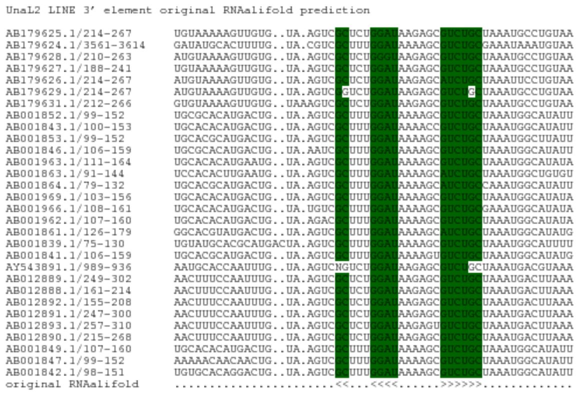

Reference Alignment of UnaL2 LINE 3' element

Reference Structure of UnaL2 LINE 3' element

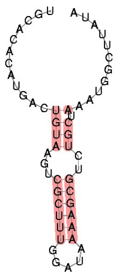

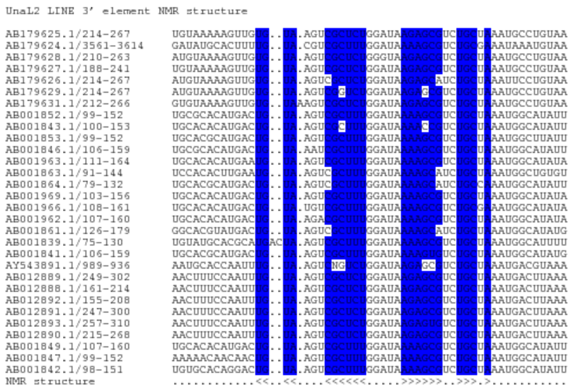

Alignment of UnaL2 LINE 3' element, RIBOSUM structure

Structure using RIBOSUM `RNAalifold`

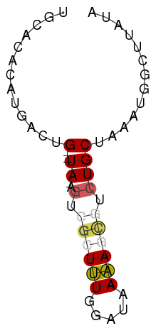

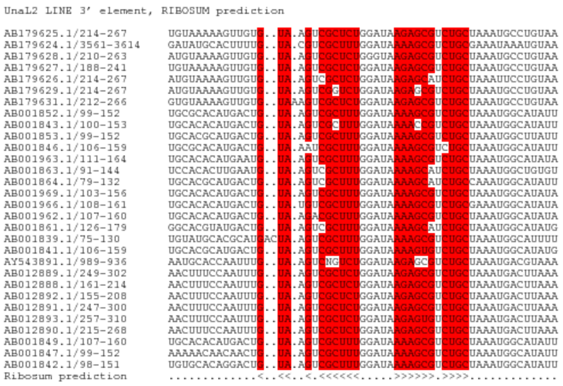

Supplement: Additional file 10 — UnaL2 LINE 3' element structure. Analysis of the effects leading to better prediction of the UnaL2 LINE 3' element structure. [file 1471-2105-9-474-S10.pdf]
